# Sensitive detection of *Bacillus thuringiensis* Cry1B toxin based on camel single‐domain antibodies

**DOI:** 10.1002/mbo3.581

**Published:** 2018-02-24

**Authors:** Wenjing Zhong, Guanghui Li, Xiaolu Yu, Min Zhu, Likun Gong, Yakun Wan

**Affiliations:** ^1^ CAS Key Laboratory of Receptor Research Shanghai Institute of Material Medical Chinese Academy of Sciences Shanghai China; ^2^ University of Chinese Academy of Sciences Beijing China

**Keywords:** biotin–streptavidin system, Cry1B, nanobody, phage display, sandwich ELISA

## Abstract

*Bt* Cry1B toxin, a residue in insect‐resistant transgenic plants, has been identified to be harmful to human health. Therefore, it is urgent to detect the Cry1B toxin level in each kind of transgenic plant. Nbs, with prominently unique physiochemical properties, are becoming more and more promising tools in the detection of target antigens. In this study, an immune phage display library that was of high quality was successfully constructed for the screening of Cry1B‐specific Nbs with excellent specificity, affinity, and thermostable. Subsequently, a novel sandwich ELISA for Cry1B detection was established, which was based on the biotin–streptavidin system using these aforementioned Nbs. This established detection system presented a linear working range from 5 to 1000 ng ml^−1^ and a low detection limit of 3.46 ng ml^−1^. The recoveries from spiked samples were in the range of 82.51%–113.56% with a relative standard deviation (RSD) lower than 5.00%. Taken together, the proposed sandwich ELISA would be a potential method for the detection of Cry1B toxin in transgenic *Bt* plants specifically and sensitively.

## 1 INTRODUCTION

There have been remarkable social and economic benefits produced by crystalline (Cry) transgenic plants since Hilder et al. reported the first genetically modified (GM) crops. Due to the momentous insect‐resistant effects, the genes of Cry toxins have been widely applied in transgenic rice, corn, and cotton by transgenic technology. Cry toxins, crucial components of GM crops, were produced by the *Bt* during sporulation and include Cry1Ab, Cry1Ac, Cry1B, Cry1C, Cry1F, Cry2Ab, Cry3A, Cry3B, and Cry9C, etc. (Bravo et al., 2013). Among these Cry toxins, Cry1B which exhibits specific activity against *lepidopterans* and *coleopterans* has been widely utilized as a biological pesticide for pest control (Crickmore et al., 1998). A series of *Bt* Cry toxins were detected in the transgenic plants, unfortunately, their residues might be harmful to human health, change the soil microbial ecological structure, and drug resistance (Conner, Glare, & Nap, 2003). Therefore, as for the content of each *Bt* Cry toxin in each GM crop, it is an issue of great importance for scientists to control and monitor.

With years of development, multiple detection assays have been developed for the accurate determination of Cry toxins. A previous study has demonstrated that polymerase chain reaction (PCR) can be utilized for the exploration of the quality as well as the quantity of specific Cry genes (Martinez, Ibarra, & Caballero, 2005), whereas, it is costly and time‐consuming despite its high sensitivity. Other approaches include microarray technology (Letowski, Bravo, Brousseau, & Masson, 2005) and mass spectrometry (MS) (Ranasinghe & Akhurst, 2002). However, the potential for the detection, identification, and quantification of Cry toxins remains to be further investigated. Alternatively, enzyme‐linked immunosorbent assay (ELISA), particularly the sandwich ELISA, has been proven to be the most convenient and widely used method for the qualitative and quantitative detection of the target proteins (Trucksess, 2001). It is not necessary for samples to be purified before ELISA analysis, a benefit for sera samples or tissue extracts which are difficult to purify. Most importantly, the development of superior antibody engineering technology provides us a great opportunity to generate novel antibodies with high affinity and specificity for ELISA analysis.

The variable domain of the heavy chain (VHH), also known as Nanobody^®^ (Nb), is the smallest recombinant antigen‐binding domain that can be conveniently produced. The unique properties of Nbs were small size and high stability making it more efficient and suitable for the application in diagnosis in comparison with conventional monoclonal antibodies and other recombinant fragments. Unlike other conventional antibodies, Nbs are able to reach and bind to the clefts and pockets on the surface of antigens due to their small size (Chakravarty, Goel, & Cai, 2014). Furthermore, Nbs can be easily cloned and expressed with high yield in heterologous systems by a reproducible manner on account of having only one domain (Rocchetti, Hawes, & Kriechbaumer, 2014), meanwhile, it is convenient for them to be modified with other materials, such as gene probes, fluorescent agents, biosensors, and radioisotopes, etc. (Saerens et al., 2005; Wang et al., 2016). In addition, Nbs are characterized by preeminent solubility/stability, nonphysiological pH, fast blood clearance, and high affinity in nanomolar or even picomolar range (Baral, Mackenzie, & Arbabi Ghahroudi, 2013). Nbs are becoming more and more promising tools for the diagnosis and treatment in biological and medical fields. In our previously reported studies, several kinds of immune assays have been established to detect Cry1Fa, Cry1Ac, and Cry1C toxins based on camel VHHs (Liu et al., 2016; Wang et al., 2014; Zhou et al., 2016).

In this study, we reported the generation of Cry1B Nbs with outstanding specificity, affinity, and thermal‐stable via phage display technology and other analytical methods. Furthermore, a sandwich ELISA assay was established based on these Nbs for the detection of Cry1B toxin in practical applications sensitively and specifically.

## 2 MATERIALS AND METHODS

### 2.1 Bactrian camel immunization and Anti‐Cry1B Nbs identification

The construction and selection of VHH phage‐displayed library were carried out as described previously (Li et al., 2015). In brief, a healthy Bactrian camel was injected with a mixture of Cry1B antigen and Freund's complete adjuvant (v: v = 1: 1) for the first time, and injected with the same volume of Freund's incomplete adjuvant for the remaining six times. After seven times of immunization, the blood was collected followed by RNA extraction and reverse transcription. Gene fragments of the VHH were amplified via two‐step nest PCR. The final gel‐purified PCR fragments (~400 bp) and the pMECS phagemid vector, which were digested with *Pst* I and *Not* I, were ligated by T4 DNA ligase to generate pMECS‐VHH recombinant phagemids before the electro‐transformation of pMECS‐VHH recombinant phagemids into *Escherichia coli* TG1 cells. Subsequently, the transformants were planted onto plates when they were enriched enough in the growth medium. Finally, the library size we constructed was estimated by counting the colony number.

Library biopanning was conducted to screen Nbs against Cry1B after library construction. A 96‐well plate was coated with Cry1B which was diluted in 100 mmol L^−1^ NaHCO_3_ (pH 8.2) (20 μg well^−1^) at 4°C overnight, and NaHCO_3_ buffer was used as control. On the next day, 200 μl of 0.1% casein was added and incubated for 2 hr to block the residual antigen‐binding sites. After the blocking and removal of the buffer, the phages were added and incubated at room temperature for 1 hr. The Cry1B‐specific binding phages were eluted by 100 mmol L^−1^ triethylamine and neutralized with 1.0 mol L^−1^ Tris‐HCl (pH 7.4), thereafter, they were used to infect TG1 cells which were diluted at various concentrations before. One hr later, the number of positive clones was compared to that of control clones by plating the infected TG1 cells into plates which contained solid LB medium and 100 μg ml^−1^ ampicillin, thus determining the fold of enrichment. It was enough for the Cry1B‐specific phages to be utilized for the following researches after amplification for two rounds of panning. Eventually, the identification, expression, and purification of soluble Nbs were performed.

### 2.2 Cross‐reactivity analysis

In order to characterize the specificity of the three selected Nbs to Cry1B, six different types of Cry toxins were chosen to test the potential cross reactivity. Samples of purified Cry toxins were generously provided by Dr. Cris Oppert and Dr. Juan Luis Jurat‐Fuentes (Department of Entomology and Plant Pathology, University of Tennessee, Knoxville, USA). Toxins (100 μl of 2 μg ml^−1^ stock solutions in 100 mmol l^−1^ NaHCO_3_) were coated on a Maxisorp plate (Thermo Scientific NUNC, Denmark). NaHCO_3_ buffer was added into wells as a control. After washing with PBST followed by blocking with 2% BSA, 100 μl of the Cry1B Nbs (2 μg ml^−1^) was added into each well and incubated for 1 hr. The above process was performed as described previously for PE‐ELISA screening (Gong, Zhu, Li, Lu, & Wan, 2016). Assays were replicated thrice.

### 2.3 Affinity analysis

Surface plasmon resonance imaging (SPRi) binding assays were performed in accordance with our previously reported study (Yan et al., 2015). Briefly, each of the Cry1B‐specific Nbs was spotted and immobilized on the NanoCapture 3D‐chip surface for 1 h. Afterward, the chip was incubated in 2 ml of 1 mol L^−1^ ethanolamine‐HCl (pH 8.5) for 20 min in order to block the activated NHS groups. The chip was loaded into a PlexArray^®^ HT analyzer (Plexera), and PBST was used as running buffer at a constant flow rate of 3 μl s^−1^ for setting up the assay. Seven serial PCT dilutions (1, 3, 9, 27, 81, 243, and 729 nmol L^−1^) were sequentially injected at a flow rate of 2 μl s^−1^. Finally, the chip was regenerated with 1:200 (v/v) H_3_PO_4_ at a flow rate of 3 μl s^−1^. Assays were conducted under the same conditions throughout the experiment. All the binding data were analyzed using the Plexera Data Analysis Module. Binding curves were fitted to a 1:1 Langmuir binding model for the illumination of the binding kinetics.

### 2.4 Thermal fluor stability test

The thermo fluor experiment was adopted to detect the Tm values of Nb2 and Nb3. A 96‐well microplate was used in the measurement, with each well containing 19.5 μl of Nb2 or Nb3, which was diluted into 500 μg ml^−1^ in triplicate and 0.5 μl of SYPRO Orange (1:125 in water). The plate was heated in a LightCycler^®^ 96 Real Time PCR Detection System (Hoffmann‐La Roche Ltd., Basel, Switzerland) from 25°C to 98°C in increments of 1°C, with an equilibration time of 10 s at each temperature. The wavelengths for excitation and emission were 490 nm and 575 nm, respectively. Eventually, the data were analyzed by software Light Cycler 96.

### 2.5 ELISA assessment for spiked samples

The proposed sandwich ELISA assay was used for the determination of Cry1B toxin in corn, wheat, and soybean samples. Dried corn, wheat, or soybean powder samples (1 g) were spiked with Cry1B toxin at five different concentrations (0.5, 1.0, 2.5, 5.0, and 10.0 mg kg^−1^), afterward, 10 ml of the protein extraction solution (0.1 mol L^−1^ PBS containing 0.1% BSA and 0.05% Tween‐20) was added. After incubating in a shaker at room temperature for 2 hr, the suspensions were collected and centrifuged at 10,000*g* for 10 min. Then the supernatants were used for sample analysis by ELISA. Each concentration was replicated three times.

## 3 RESULTS AND DISCUSSION

### 3.1 Generation of an immune library against Cry1B

At present, technologies of phage surface display (Johnsson & Ge, 1999), yeast surface display (Boder & Wittrup, 2000), and ribosome display (Yan & Xu, 2006) are classic methods which are being widely adopted for the screening of antigen‐specific Nbs. Among them, phage surface display is the fastest and lowest‐cost for Nb screening. To obtain Cry1B‐specific Nbs, a young healthy camel was immunized with pure Cry1B‐His protein for seven times for the achievement of a satisfactory immune response.

Afterward, we successfully constructed a high‐quality phage display library according to the procedures which were illustrated by one of our previously reported studies (Gong et al., 2016) with the size of 1.6 × 10^9^ colony‐forming units (CFU) (data not show). Meanwhile, the insertion rate of the library was evaluated by PCR through randomly picking out 24 individual clones, which was eventually proved to be approximately 100% (data not show). Taken together, these above results demonstrated that an anti‐Cry1B phage library which was of high quality was successfully constructed.

### 3.2 Identification of Cry1B‐specific Nbs

**Figure 1:**
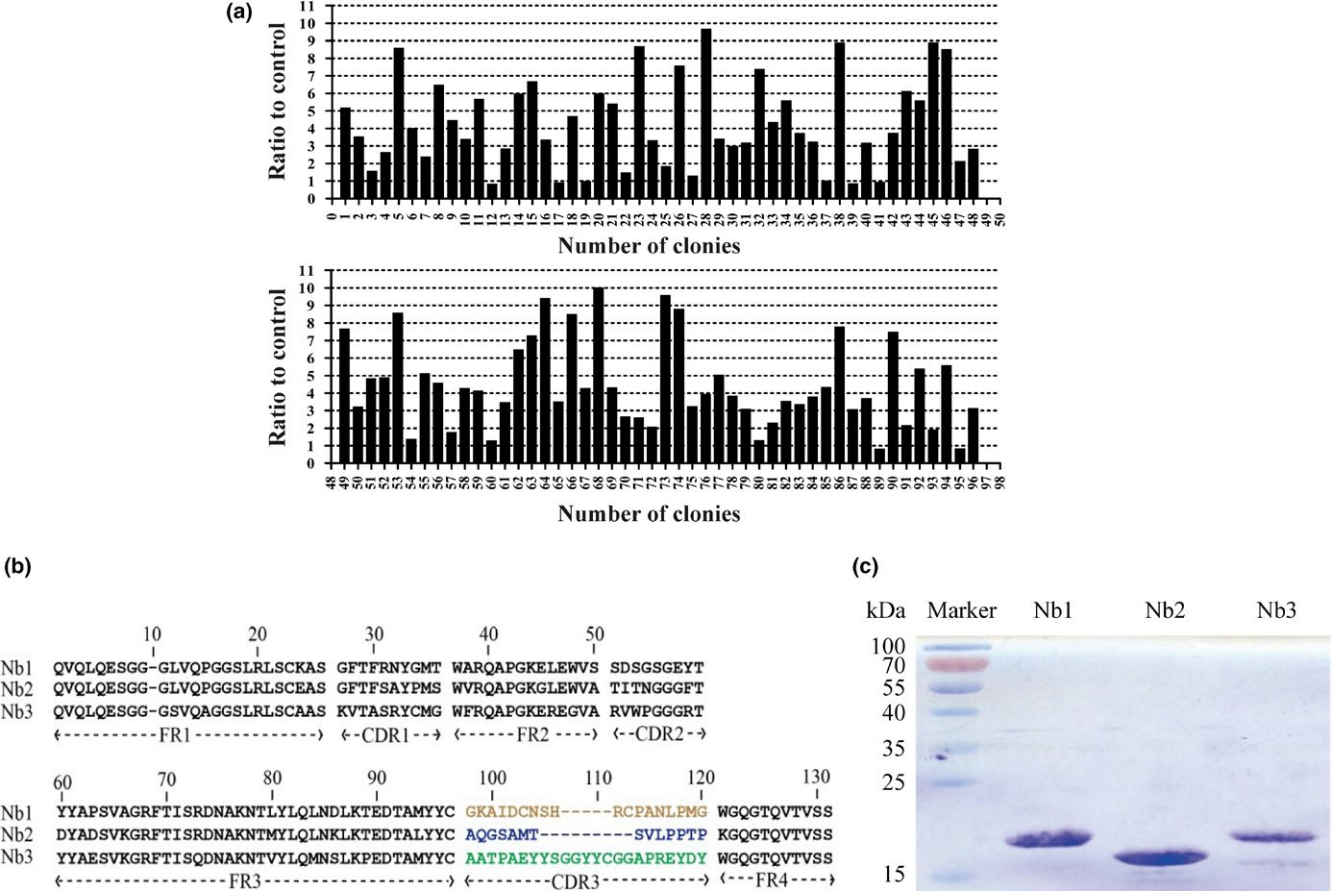
Identification of Cry1B‐specific Nbs. (a) 68 positive clones (ratio ≥3) were selected from PE‐ELISA. (b) Three kinds of different amino acid sequences of anti‐Cry1B Nbs were identified. (c) Three Nbs having different sequences were purified

After constructing the Cry1B‐specific library, phage display technology was used to screen the Cry1B‐specific Nbs. Firstly, the differences in relative enriching efficiency of phage particles eluted from wells between control and those coated with Cry1B were analyzed. Next, periplasmic extraction ELISA (PE‐ELISA) was carried out to detect the positive clones among the randomly selected 96 individual colonies. Fortunately, 68 of 96 colonies were identified as positive, and the binding ratio of positive colonies versus negative control was more than 3 (Figure 1a), with the last clone acting as a blank control. Thereafter the positive colonies were sequenced and analyzed. Finally, the selected VHHs were categorized into three groups according to the diversity of their amino acid sequences in CDR3 (Figure 1b). Three Nbs named as Nb1, Nb2, and Nb3, respectively, were selected from the above three groups and applied in further researches.

### 3.3 Expression and purification of the isolated Nbs

Previous procedures for Nb expression required cloning of VHH genes into another vector and transforming of the recombinant vector into an Escherichia coli strain, such as BL21 or PAX51 (Ladenson, Crimmins, Landt, & Ladenson, 2006). Herein, we have bypassed these steps and directly transformed pMECS‐containing VHH genes from TG1 to WK6 cells for the expression of VHH‐HA‐His6 fusion protein. The HA‐tag could be used as a recognition component in ELISA and His6 could be used as a tag for Nbs purification. Sodium dodecyl sulfate‐polyacrylamide gel electrophoresis (SDS‐PAGE) analysis demonstrated that high quality of Nbs with the purity of more than 90% (Figure 1c) were obtained by this optimized procedure.

### 3.4 Biotinylation of the Nbs for Cry1B capture

As for the detection in ELISA, a primary Nb and a secondary Nb were needed to capture the antigen and target antigen that was conjugated with Horseradish Peroxidase (HRP), respectively. Therefore, it is important to identify the binding epitopes of three Nbs on Cry1B, which were authenticated by antibody paring assay (data not shown). Among the three Cry1B‐specific Nbs, Nb1, and Nb2 recognized the same epitope while Nb3 recognized a different one. According to the result of epitope mapping and specificity, Nb2 and Nb3 were chosen for the conducting of immunoassay.

Biotin–streptavidin (SA) system was one of the most widely used conjugation pairs in immunoassays, which promoted the analytical sensitivity with multiple amplification effects (Pawlak, Moyer, & Polarek, 1987). Nb2 and Nb3 were valuable elements in the Biotin‐SA System, therefore, one was modified with biotin and the other with HRP (Yan, Li, Hu, Ou, & Wan, 2014). According to the results of paired test, Nb2 was chosen to conjugate with biotin, taking the advantage of the containment of biotin acceptor domain (BAD) for labeling in plasmid pBAD17. Biotin molecules were conjugated with BAD in a reaction catalyzed by a ligase which was encoded by plasmid pBirA. Nb2 was biotinylated for further application in the Biotin‐SA System.

### 3.5 Analysis of specificity, affinity, and thermal‐stable

**Figure 2:**
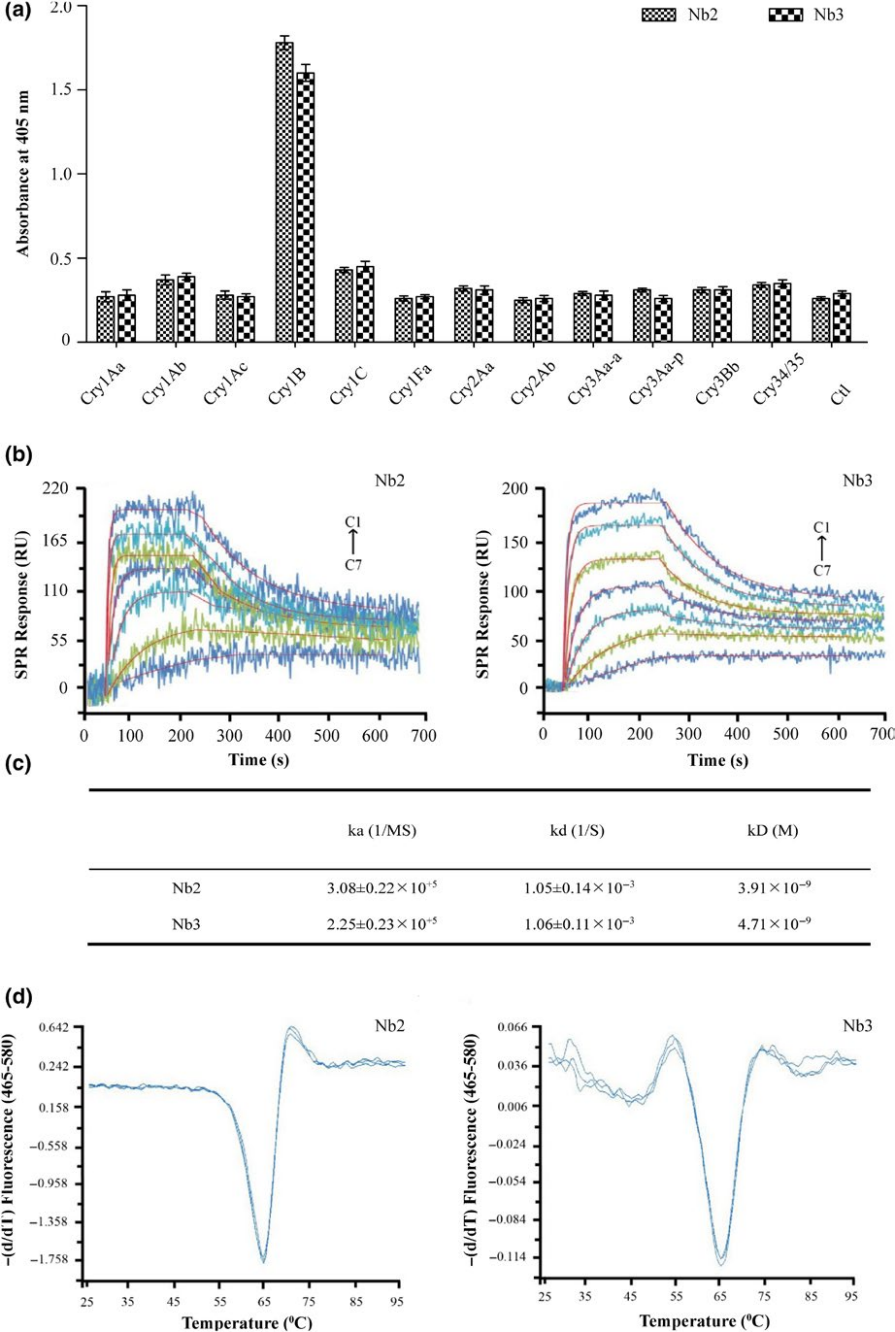
Feature analysis of purified Cry1B Nbs. (a) The specificity of purified Cry1B Nbs was tested by indirect ELISA with different types of Cry toxins. (b) The equilibrium dissociation constants between Cry1B and the paired Nbs (Nb2 and Nb3) was measured with the kinetic analysis by SPRi. Cry1B dilutions were injected at concentrations of 1, 3, 9, 27, 81, 243, and 729 nmol L^‐1^ (C7–C1). (c) Relevant parameters of kinetic analysis. (d) The thermal stability of Nb2 and Nb3 was measured by a fluorescence‐based assay with Sypro Orange dye. The temperature was set from 25 to 98°C in increments of 1°C and three repeats were performed

The specificities of the isolated Nbs to Cry1B were also verified. ELISA assays were performed by coating the Maxisorp plate with equivalent concentration of alternative Cry toxins, by which high specificity of the two Nbs to Cry1B as well as no cross‐reactivity with any other Cry toxins was confirmed (Figure 2a). These results indicated that the obtained Nbs were highly specific to Cry1B toxin.

As the crucial characteristic of an antibody, affinity in each of these two paired Cry1B‐specific Nbs was measured by surface plasmon resonance (SPR) binding assay. The sensor grams demonstrated that the equilibrium dissociation constants (kD) of the two Nbs were 3.41×10^−9^ mol L^−1^ and 4.73×10^−9^ mol L^−1^ (Figure 2b,c), respectively. Compared to other antibodies for Cry toxins, these two Nbs showed relatively higher affinity (Li, Zhu, Zhang, Liu, & Wan, 2014).

Nbs have exhibited great stability even under stringent conditions, such as thermal denaturation (Liu et al., 2016). A fluorescence‐based thermal‐stable assay with Sypro Orange dye was used to determine the thermal‐stable of the purified Cry1B‐specific Nbs. In brief, the results indicated that the average value of melting temperature (Tm) of both Nb2 and Nb3 was 65°C (Figure 2d).

### 3.6 Determination of Cry1B based on a sandwich ELISA

**Figure 3:**
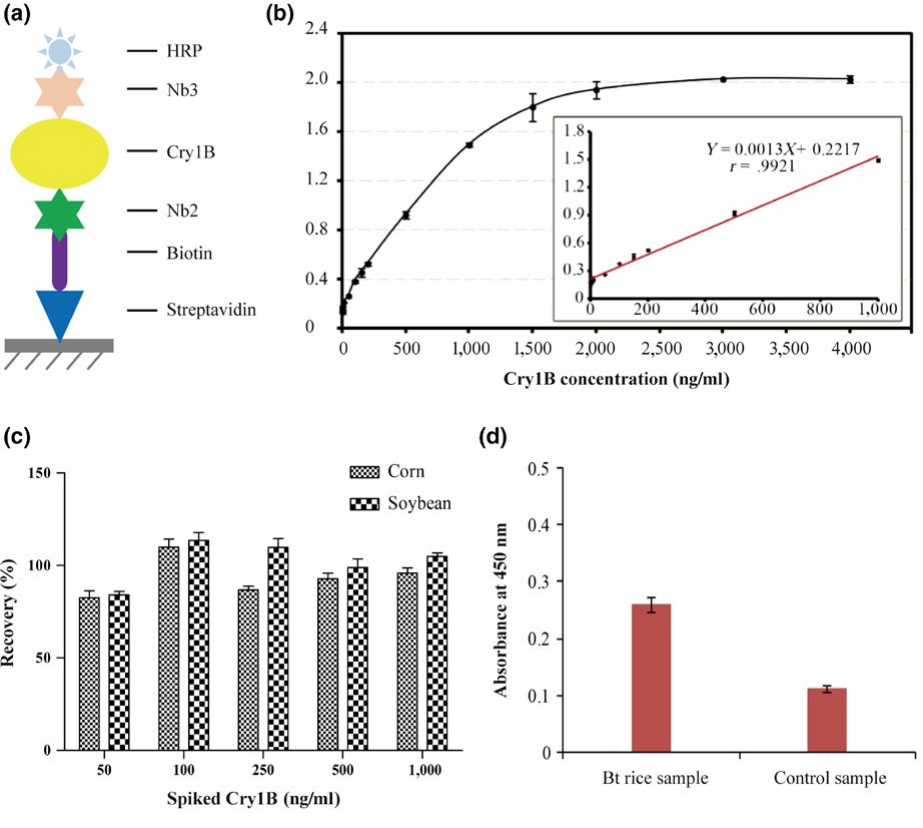
Cry1B detection by the improved sandwich ELISA and recoveries study. (a) Outline of strategies to detect Cry1B by sandwich ELISA based on biotin–streptavidin system. (b) Calibration curve for Cry1B detection. The linear relationship between the absorbance at 450 nm and the Cry1B concentration was in the range from 5 to 1,000 ng ml^‐1^. (c) Recoveries of Cry1B toxin from spiked samples. (d) Determination of real Bt rice sample. The leaves from a Bt rice that could express Cry1B toxin and the corresponding nontransformed rice leaves used as control were detected by the proposed sandwich ELISA

Based on the conventional sandwich ELISA, an improved ELISA for Cry1B detection was developed and established using of the Biotin‐SA System. In this assay, Nb2 functioned as the capturer, and Nb3 modified with HRP acted as the detector (Figure 3a). The calibration curve showed a good linear correlation (*r* = .9921) between the absorbance at 450 nm and the Cry1B concentrations in the range of 5–1,000 ng ml^−1^ (Figure 3b). This linear range was broader and more sensitive for Cry1B detection than that of the indirect competitive ELISA which adopted scFv (range of 190–1,100 ng ml^−1^). The limit of detection (LOD) of the improved ELISA was calculated in accordance with the recommendations provided by International Union of Pure and Applied Chemistry (IUPAC), that is, LOD = 3×Sb/b, where Sb was the standard deviation (*n* = 3) of the blanks, and b was the slope value of the respective calibration graph. In our assay, Sb was 0.0015 and b was 0.0013, giving a LOD of 3.46 ng ml^−1^ and illustrating a high sensitivity. This evaluation also indicated that the LOD of Nbs was lower than those of other conventional sandwich ELISAs, which detected Cry1Ab based on a rabbit polyclonal antibody and a scFv (8 ng ml^−1^) (Zhang et al., 2014). Taken together, it was evidenced that this method could be applied in the detection of Cry1B with a great promise.

### 3.7 Recoveries study

To test the applicability of the developed ELISA for the detection of Cry1B in food samples, corn and soybean samples were spiked with Cry1B at different concentrations and detected by the proposed sandwich ELISA. The recoveries of Cry1B in the spiked samples ranged from 82.51% to 113.56%, with a RSD ranging from 1.65% to 4.77% (Figure 3c), suggesting the reliability and accurateness of the proposed ELISA for the quantitative determination of Cry1B toxin in plant/food samples.

Thereafter, the leaves from *Bt* rice (expressing the cry1B gene) and the corresponding nontransformed rice were detected. As presented in Figure 3d, the absorbances of the *Bt* rice sample and the control rice sample were 0.25 and 0.13, respectively, suggesting the potential application of our proposed sandwish ELISA in the determination of the presence of *Bt* Cry1B in transgenic rice.

Until now, mounting evidences about detection of various Cry toxins (Cry1Ac, Cry1Ie, Cry1C, Cry1Fa) base on Nbs have been published (Liu et al., 2016; Wang et al., 2014; Xu et al., 2017; Zhou et al., 2016). Among them, a series of papers about Cry1Ac, Cry1C, and Cry1Fa came from our laboratory. In this work, we demonstrated the efficient, specific and sensitive detection of Cry1B with Nbs by a sandwich ELISA assay. The results showed that the linear working range was from 5 to 1,000 ng ml^−1^ with a low limit detection of 3.46 ng ml^−1^. The recoveries in the spiked samples were 82.51%–113.56%, with a RSD lower than 5.00%. These findings indicated that the Nbs‐based sandwich ELISA would become a reliable tool of great promise for the detection of Cry1B in agricultural and environmental samples in the future.

## CONFLICT OF INTEREST

The authors declare that there are no conflicts of interest.
